# The management of abdominal hydatidosis after the rupture of a pancreatic hydatid cyst: a case report

**DOI:** 10.1186/1752-1947-9-27

**Published:** 2015-02-10

**Authors:** Victoria Bîrluţiu, Rareş Mircea Bîrluţiu

**Affiliations:** 10 Faculty of Medicine Sibiu, Academic Emergency Hospital SIBIU – Chief of the Infectious Diseases Clinic, “Lucian Blaga” University Sibiu, Sibiu, Romania; 11 Faculty of Medicine Sibiu, “Lucian Blaga” University Sibiu, Alba-Iulia Str. No.79 23/8, Sibiu, 550052 Romania

**Keywords:** Anti-parasite treatment, Echinococcosis, *Echinococcus* genus, Pancreatic cyst

## Abstract

**Introduction:**

Echinococcosis or hydatidosis is a zoonosis caused by cestodes from the genus *Echinococcus*; its habitat is the small intestine of the definitive host, represented by dogs/carnivorous animals, where it produces eggs which are eliminated in the environment. Cystic echinococcosis represents more than 95% of the hydatidosis cases registered annually. The most frequent localization is the hepatic one, followed by the pulmonary localization with a ratio of 2.5:1. A pancreatic localization represents 0.2% of hydatidosis cases with a higher possibility of disseminating intra-abdominally. The incidence of hydatidosis in Romania has not been investigated yet through national studies.

**Case presentation:**

We present the case of a 54-year-old Caucasian man who underwent emergency surgery in 1989 for symptoms suggestive for an acute abdomen. He was diagnosed intraoperatively with rupture of a pancreatic hydatid cyst, having a caudal localization and complicated by necrotic acute pancreatitis. Our objective is to describe a patient with hydatidosis, with unfavorable evolution after two surgical interventions, with intra-abdominal dissemination, for whom we considered the best therapeutic choice to be long-term anti-parasite drugs.

**Conclusions:**

He has been treated with albendazole for 6 years and he shows a very good tolerance; praziquantel (600mg/week) was also administered and he is under clinical and biological screening. There is no general consensus on the duration of anti-parasite treatments.

**Electronic supplementary material:**

The online version of this article (doi:10.1186/1752-1947-9-27) contains supplementary material, which is available to authorized users.

## Introduction

Echinococcosis or hydatidosis is a zoonosis caused by cestodes from the genus *Echinococcus*; its habitat is the small intestine of the definitive host, represented by dogs/carnivorous animals, where it produces eggs that are eliminated in the environment. Intermediate hosts, represented by goats, sheep, pigs and horses, ingest the eggs. Eggs can be present in humans, for example after the consumption of raw meat; the hydatidosis will be located in the liver or lungs and more rarely in the brain, heart, pancreas, kidney or bones. The most frequent localization is the hepatic one, followed by the pulmonary localization with a ratio of 2.5:1 [1]. Pancreatic localization represents 0.2% of hydatidosis cases [2]. In humans, hydatidosis can manifest itself in three forms: cystic echinococcosis, alveolar echinococcosis and polycystic echinococcosis, diagnosed in Central and South America [3]. It is considered that the disease is endemic in Africa, Alaska, Europe – Iceland, Austria, Germany, Switzerland – with people who work in agriculture [4, 5], in South America and Australia. Cystic echinococcosis represents more than 95% of the hydatidosis cases registered annually; there is estimated to be 2 to 3 million cases of cystic echinococcosis [6]. The incidence of hydatidosis in Romania has not been investigated yet through national studies, but a higher risk is admitted in the pasture areas in the South and Central regions of the country.

## Case presentation

**Figure 1:**
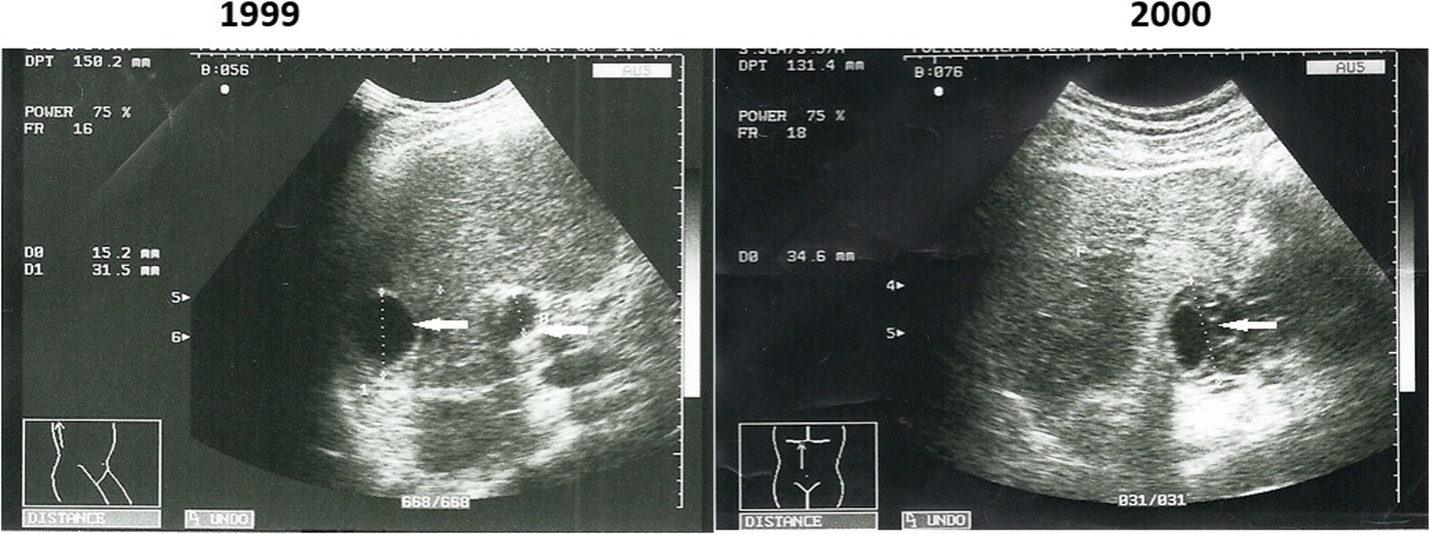
**Ultrasound images after initial diagnostic.**

We report the case of a 54-year-old Caucasian man who was admitted to our Infectious Diseases Department in 2008, 1 month after discharge from the Surgery Department for treatment of abdominal hydatidosis. At admission, he presented fever, shivering, jaundice, hyperchromic urine, and a poor general condition. He was diagnosed with a hydatid cyst in 1989, when he presented to an emergency room with symptoms suggestive for an acute abdomen; he was then suspected to have a perforated ulcer (lacking the possibility of imaging investigations such as computed tomography [CT], scan or magnetic resonance imaging [MRI]). He was diagnosed intraoperatively with rupture of pancreatic hydatid cyst, having a caudal localization and complicated by necrotic acute pancreatitis. A Mabit-Lagrot operculectomy was performed, with double drainage of the cystic cavity and subhepatic drainage. His postoperative evolution was associated with a febrile episode, a progressive deterioration of his nutrition status and the appearance of a pure pancreatic fistula with a debit of 700 to 800mL/day and later an enterocutaneous fistula between the transverse colon and the skin surface appeared, due to the erosion of its walls by the pancreatic secretions. Subsequent to a steady treatment with parenteral nutrition, antibiotherapy (ampicillin) and adequate local treatment (sequestrectomy, lavage and lactic acid solution aspiration, 4.5% –Trémolières method), his evolution was slowly favorable. The treatment resulted in the closure of the stercoral fistula and the healing *per secundam* of the surgical wound, but two fistulous orifices remained: one in his epigastrium and the other one in his right hypochondria, through which a creamy yellowish pus was eliminated and where *Escherichia coli* could be isolated. A fistulogram revealed a double fistulous trajectory, ending in a pancreatic cavity with anfractuous walls. He was released 73 days later, with a reduced debit of the fistula of circa 10mL; he recovered slowly and favorably with the closure of the fistula within the next 2 months. Two years after the operation he was diagnosed with diabetes type II, which was counterbalanced with oral antidiabetic medication; he also had recurrent episodes of abdominal pain, which were labeled as biliary colic and ameliorated by anti-spastic medication. Seven years after the surgery, an ultrasound evaluation and an abdominal CT scan revealed a multi-septated cystic image of 10×5cm, involving the pancreatic body and tail, dilated intrahepatic biliary tracts and two small cystic images in the liver: segments VI and VII (Figure 1).

In 2002 he experienced an anaphylactic shock resulting from a fissure of an abdominal cyst. He was admitted with violent epigastric pain, malaise, itching, dyspnea, altered deglutition, and severe hypotension; during this admission he was treated with epinephrine, fluids, oxygen, antihistaminic drugs, and corticotherapy. In 2007 he was admitted with pains in his right hypochondria radiating in his right shoulder, nausea, anorexia and loss of weight. An abdominal CT scan revealed multiple hydatid cysts in both his hepatic lobes, with partially calcified walls; some of the cysts had multiple septations or detached laminated membranes, without excluding a fissure (no contrast-enhancing agent was administered due to the patient's allergic history). One of the cysts was 7cm in diameter; it was localized in the hepatic hilum and produced a compression effect with a slight dilatation of the intrahepatic ducts predominantly in his left hepatic lobe. His gallbladder appeared hydropic with small slight hyperdense images in the interior. A gigantic polycyclic and polyseptate image was found in the hypogastric area; its walls were partially calcified and the contents predominantly fluid, with a transversal diameter of 17cm, reaching his spleen: sonographic findings consistent with a peritoneal hydatid cyst. The pancreatic head appeared enlarged with preserved structure and no ascites was identified. Surgery revealed the presence of an intense process of perivisceritis of multiple hepatic hydatid cysts (segments IV, V, VI and VII), caudal pancreatic hydatid cyst and peritoneal hydatid cysts, of which one was subhepatic and the other retrogastric, and chronic lithiasic cholecystitis. An exploratory laparotomy was performed, lysis of adhesion, anterograde operculectomy, multiple peritoneal drainage (right subphrenic, subhepatic and in the Pouch of Douglas), drainage of the remaining cavity at the level of the hydatid cyst in the omental bursa and at the level of the pancreatic hydatid cyst.

In his postoperative state he developed an *Enterococcus* species over-infection of the remaining cavity of the hydatid cyst in the omental bursa and of the surgical wound, with a slowly favorable evolution under treatment with teicoplanin for 14 days.

An abdominal ultrasound examination performed postoperatively revealed high caliber of his portal vein (16mm), with a narrow lumen and intraluminal thrombosis, his common bile duct of 10mm and fluid in the peritoneal cavity.

At admission in our department, during the pre-admission examination, he was febrile (38°C), had discreet jaundice, with non-altered respiration, heart rate (HR) 86/minute, blood pressure (BP) 125/70mmHg, with pain in his upper abdominal level and a hepatomegaly. Laboratory examinations revealed the following alterations: hemoglobin 12.5g/dL, hematocrit 37.9%, leucocytes 4720/mm^3^, granulocytes 60.2%, lymphocytes 17.6%, monocytes 9.1%, basophiles 0.4%, eosinophils 12.7%, glucose 130mg/dL, aspartate aminotransferase 29U/L (reference values 20 to 42), alanine aminotransferase 40U/L (references values 20 to 43), erythrocyte sedimentation rate 64mm/hour, gamma-glutamyl transferase 108U/L, bilirubin 1.46mg/dL, conjugate bilirubin 0.72mg/dL, international normalized ratio 1.82, activated partial thromboplastin time 29.2 seconds, total proteins 8.4g/dL, albumins 43.5%, α1-globulins 2.9%, α2-globulins 10.5%, β-globulins 9.9%, γ-globulins 33.2%, and albumins/globulins ratio 0.77.

Under antibiotic treatment (ceftazidime 4g/day), anti-parasite drugs (albendazole) 2×400mg/day, ursodeoxycholic acid 3×250mg/day and proton-pump inhibitors, his evolution was favorable. Due to the risks of unpredictable evolution of the hydatid cysts, it was suggested to continue the maintenance treatment with albendazole (800mg/day) and ursodeoxycholic acid 3×250mg/day.

His evolution under treatment was favorable during the next 3 years, not requiring the cessation of the anti-parasite therapy and his biological balance was within normal limits, except for hyperglycemia (168mg/dL) and hypergammaglobulinemia (24.5%).

In 2011, he was admitted with a sudden start of psychomotor agitation, abdominal pains, profuse sweating, hiccups, irritation cough, skin rash, and hyperchromic urine. An examination revealed a poor general state, jaundice, scraping lesions on both legs, diminished pulmonary right basal vesicular murmur, rhythmic cardiac sounds, HR 88 beats/minute, BP 110/70mmHg, sensitive abdomen during palpation in his epigastric region and his right hypochondria, hepatomegaly, splenomegaly, anxiety, psychomotor agitation, with no signs of meningeal irritation. Laboratory examinations revealed the following alterations: platelets 147000/mm^3^, glucose 160mg/dL, gamma-glutamyl transferase 90U/L, total bilirubin 1.70mg/dL, total proteins 7.4g/dL, γ-globulins 24.4%, and albumins/globulins ratio 0.79.

**Figure 2:**
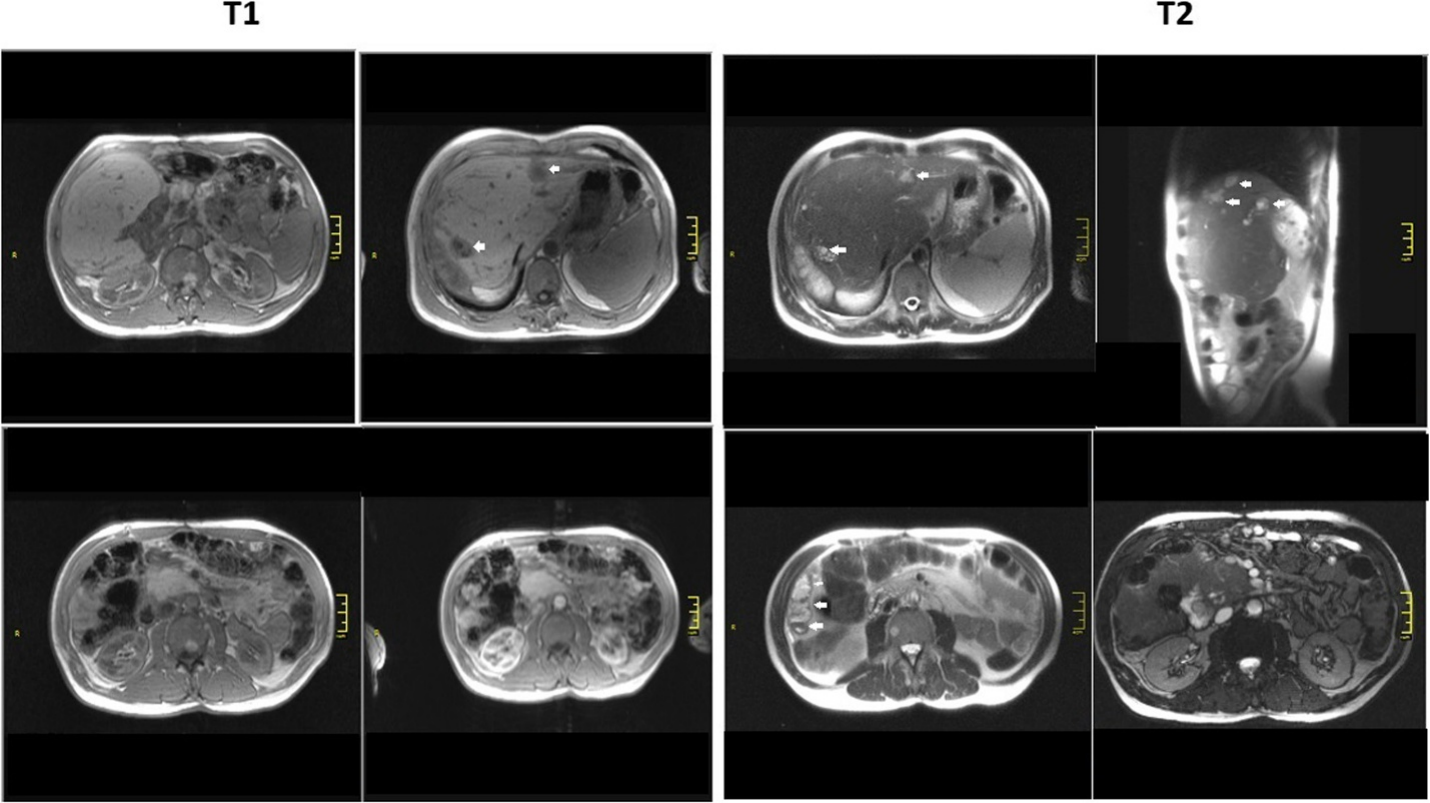
**Magnetic resonance imaging from 2011.**

A pulmonary radiography revealed an emphatic bilateral peribronchovascular interstitium. A cranium CT scan was performed in order to exclude the presence of any intracerebral hydatid cysts (a moderate cortical atrophy was found) and a transthoracic echocardiography which excluded the localization of *Echinococcus granulosus* at this level. An abdominal MRI (Figure 2) revealed nine findings. First, hepatomegaly with multiple images of partially fluidic pseudocysts/abscesses resulting in the compression of his left hepatic biliary tracts. Second, fluid collection posterior to the 7th hepatic segment: pseudocysts or abscesses. Third, cephalic hypertrophy of his pancreas with hypovascular zones on the inside: small necrosis, the head and the tail of his pancreas were not clearly delimited but some cystic images up to 30mm could be seen at this level. Fourth, partial thrombosis of the portal vein; it appeared dilated and only partially loaded when a contrast-enhancing agent was administered. Fifth, subhepatic collateral circulation, retroperitoneal: cavernoma. Sixth, homogenous splenomegaly; seventh, small retroperitoneal cysts; eighth, small cortical infracentimetric cysts in his left kidney; and ninth, minimum right pleurisy.

His evolution was favorable under antibiotic treatment with meropenem 4g/day together with dexamethasone 16mg/day, proton pump inhibitors and albendazole 800mg/day. In 2014 it was decided to associate praziquantel 600mg/week with the albendazole 800mg/day treatment. Presently, his general state is good, with no subjective complaints and non-altered hepatic tests (Additional file 1).

## Discussion

The intraperitoneal rupture of a pancreatic hydatid cyst has a variable incidence in the literature, from 1% to 8%; the rupture of peritoneal cysts may occur in the pleural cavity, the bile ducts, and the digestive tract [7]. The rupture of peritoneal cysts in the digestive tract is not only responsible for abdominal pains, vomiting, acute abdomen, cholangitis, and sepsis but also for skin rashes, urticaria or anaphylactic shock; these manifestations (skin rashes, urticaria or anaphylactic shock) were present in our patient. Pleural and transdiaphragmatic involvement were responsible for our patient’s pains at the base of his right hemithorax, hiccups and irritation cough. Surgical procedures are avoided in peritoneal hydatidosis but most authors recommend surgical interventions in order to avoid risks of peritonitis and death [7, 8].

In the case we have reported, we witness a diffuse abdominal hydatidosis with the presence of peritoneal, pancreatic, hepatic, splenic and possible renal cysts, for which the surgical intervention does not present real benefits. In addition, the anterior surgical interventions were complicated with fistulas, over-infections, portal vein thrombosis, the extension of the abdominal hydatidosis, anaphylactic shock, and others; all these facts contributed to the difficult recovery of the patient's health status. In such a case, the only remaining therapeutic option is anti-parasite drugs. The administration of albendazole for 1 month before and after surgery, or of praziquantel for 2 weeks would have reduced the risk of anaphylaxis or of postoperative recurrences [9, 10]. It is considered that the mortality rate after the first surgical procedure is between 0.9 and 3.6%, 6% after the second one and 20% after a third one [11].

There is no general consensus on the duration of anti-parasite treatments. The World Health Organization recommends the postoperative administration of albendazole for at least 1 month or mebendazole for 3 months [12] but the risk of peritoneal recurrences determines the continuation of the treatment; the longest period described in the literature was of 1 year [13]. The case presented is of a patient for whom we decided not to repeat surgical treatment, but to administer treatment with albendazole (800g/day) for 6 years to which he showed a very good tolerance; praziquantel (600mg/week) was also associated and he has been under clinical and biological screening for 7 months.

## Conclusions

Cases of pancreatic hydatidosis affecting secondarily the liver, spleen, kidneys, peritoneum and retroperitoneal space are extremely rare; they are associated with the risk of anaphylactic shock, having a potentially fatal evolution due to the rupture of the peritoneal cysts. The management of these cases must be individualized and the decision of a long-term anti-parasite treatment is an option to be taken into consideration.

## Consent

Written informed consent was obtained from the patient for publication of this case report and any accompanying images. The study was accepted by the Ethics Committee of the hospital and they encouraged the publishing of this article. A copy of the written consent is available for review by the Editor-in-Chief of this journal.

## Electronic supplementary material

Additional file 1: **Flow diagram.** (DOCX 56 KB)

## Abbreviations

BP: Blood pressure
CT: Computed tomography
HR: Heart rate
MRI: Magnetic resonance imaging.
