# Supplementary material for: The management of abdominal hydatidosis after the rupture of a pancreatic hydatid cyst: a case report
Source: J Med Case Rep. 2015 Feb 10;9:27. doi: 10.1186/1752-1947-9-27 (PMC4429723; doi:10.1186/1752-1947-9-27)
Supplement: Supplementary file 1 — Additional file 1: Flow diagram. (DOCX 56 KB) [file 13256_2014_3156_MOESM1_ESM.docx]

**Due to the risks of evolution of the hydatid cysts and the patient’s history,**

**long-term treatment with albendazole was suggested**

One month later:

- presented himself to our clinic (Infectious Diseases Clinic)
- he started anti-parasites treatment

Hospitalized

Computed tomography and ultrasound evaluation – **multiple hydatid cysts**

Surgery

Discharged

Hospitalized

CT and ultrasound evaluation – **multiple hydatid cysts**

Surgery

discharged

Computed tomography and ultrasound evaluation

**2007**

Anaphylactic shock from a fissure of an abdominal cyst

CT and ultrasound evaluation

**2002**

CT and ultrasound evaluation

**1996**

Diagnosed with diabetes type 2

Emergency surgery for acute abdomen. Intraoperatively diagnosed with **pancreatic hydatid cyst.**

Discharged 73 days later

**1991**

Emergency surgery for acute abdomen. Intraoperatively diagnosed with **pancreatic hydatid cyst.**

Discharged 73 days later

**1989**

**BIRTH**

**Favorable evolution under treatment.**

**During this period of time the patient had carried out only follow-up examination and laboratory tests**

He was admitted to our clinic:

-laboratory tests

- ultrasound imaging, computed tomography scan and magnetic resonance imaging

- diagnosis: hydatidosis

**We continued treatment with albendazole.**

**Favorable evolution under treatment.**

**During this period of time the patient had carried out only follow-up examination and laboratory tests**

**In 2014 we associated praziquantel with his treatment**

**–** follow-up visits, patient`s general state is good, no subjective complaints.

Case report

**2011**
